# Granulocyte Colony-Stimulating Factor Accelerates the Recovery of Hepatitis B Virus-Related Acute-on-Chronic Liver Failure by Promoting M2-Like Transition of Monocytes

**DOI:** 10.3389/fimmu.2022.885829

**Published:** 2022-05-16

**Authors:** Jingjing Tong, Hongmin Wang, Xiang Xu, Zhihong Wan, Hongbin Fang, Jing Chen, Xiuying Mu, Zifeng Liu, Jing Chen, Haibin Su, Xiaoyan Liu, Chen Li, Xiaowen Huang, Jinhua Hu

**Affiliations:** ^1^Chinese PLA Medical School, Beijing, China; ^2^Senior Department of Hepatology, the Fifth Medical Center of PLA General Hospital, Beijing, China; ^3^Department of Infectious Diseases, Beijing Jishuitan Hospital, Beijing, China; ^4^Peking University 302 Clinical Medical School, Beijing, China; ^5^Laboratory of Translational Medicine, Medical Innovation Research Division of Chinese PLA General Hospital, Beijing, China; ^6^Center for Drug Evaluation, National Medical Products Administration, Beijing, China; ^7^Department of Biostatistics, Bioinformatics and Biomathematics, Georgetown University Medical Center, Washington, DC, United States

**Keywords:** acute-on-chronic liver failure, granulocyte colony stimulating factor, monocytes, hepatitis B virus, inflammation, prognosis, cytokine

## Abstract

**Background and Aim:**

Acute-on-chronic liver failure (ACLF) has a high mortality rate. The role of granulocyte colony-stimulating factor (G-CSF) in ACLF remains controversial. Monocytes/macrophages are core immune cells, which are involved in the initiation and progression of liver failure; however, the effect of G-CSF on monocytes/macrophages is unclear. The study aimed to verify the clinical efficacy of G-CSF and explore the effect of it on monocytes in hepatitis B virus (HBV)-related ACLF (HBV-ACLF) paitents.

**Methods:**

We performed a large randomized controlled clinical trial for the treatment of HBV-ACLF using G-CSF. A total of 111 patients with HBV-ACLF were prospectively randomized into the G-CSF group (5 μg/kg G-CSF every day for 6 days, then every other day until day 18) or the control group (standard therapy). All participants were followed up for at least 180 days. The relationship between monocyte count and mortality risk was analyzed. The effect of G-CSF on the phenotype and function of monocytes from patients with HBV-ACLF was evaluated using flow cytometry *in vivo* and *in vitro* experiments.

**Results:**

The survival probability of the G-CSF group at 180 days was higher than that of the control group (72.2% vs. 53.8%, *P* = 0.0142). In the G-CSF-treated group, the monocyte counts on days 0 and 7 were independently associated with an evaluated mortality risk in the fully adjusted model (Model 3) [at day 0: hazard ratio (HR) 95% confidence interval (CI): 15.48 (3.60, 66.66), *P* = 0.0002; at day 7: HR (95% CI): 1.10 (0.50, 2.43), *P*=0.8080]. Further analysis showed that after treatment with G-CSF in HBV-ACLF patients, the expression of M1-like markers (HLA-DR and CD86) in monocytes decreased (HLA-DR: *P* = 0.0148; CD86: *P* = 0.0764). The expression of MerTK (M2-like marker) increased (*P* = 0.0002). The secretion of TNF-α, IL-6, and IL-10 from monocytes decreased without lipopolysaccharide (LPS) stimulation (TNF-α: *P* < 0.0001; IL-6: *P*= 0.0025; IL-10: *P* = 0.0004) or with LPS stimulation (TNF-α: *P* = 0.0439; *P* = 0.0611; IL-10: *P* = 0.0099). Similar effects were observed *in vitro* experiments.

**Conclusion:**

G-CSF therapy confers a survival benefit to patients with HBV-ACLF. G-CSF can promote the anti-inflammatory/pro-restorative phenotype (M2-like) transition of monocytes, which may contribute to the recovery of ACLF.

**Clinical Trial Registration Number**

ClinicalTrials.gov, identifier (NCT02331745).

## Introduction

Acute-on-chronic liver failure (ACLF) is a clinical syndrome characterized by severe hepatic dysfunction resulting from acute injury to an underlying chronic liver disease and a substantially high short-term mortality rate (1, 2). The hallmark of ACLF is the large-area necrosis of liver tissue and severe inflammation. However, current treatment for ACLF focuses on targeting the triggering insult and optimizing the clinical management of complications (3). Efficacious therapeutic strategies, aimed at promoting liver regeneration and restricting inflammation, remain limited. Currently, liver transplantation is the only effective therapy to prevent ACLF; however, its application may not be generalizable because of its cost prohibitive nature and the insufficiency of donors (4). Therefore, there is an unmet need for novel treatment approaches.

Although traditionally considered a hematopoietic regulator, granulocyte colony stimulating factor (G-CSF) has been regarded as a candidate treatment for ACLF recently. Several studies have established the efficacy and safety of G-CSF in the management of ACLF (5–9). However, a recent study showed no significant survival benefit of G-CSF in individuals with ACLF (10). Therefore, more well-conducted investigations are required.

The role of G-CSF in liver tissue regeneration and in regulation of the immune response has been shown. Studies in animals and humans indicate that G-CSF can promote the migration of hematopoietic stem cells as well as the proliferation and differentiation of hepatic progenitor cells during liver failure (5, 11, 12). Other studies have demonstrated the useful effects of G-CSF on dendritic cells and neutrophils in ACLF (13, 14). However, as the core immune cells involved in the development and progression of liver failure (15–17), the effects of G-CSF on monocytes/macrophages in ACLF patients remain unexplored. Monocytes/macrophages are characterized by high diversity and plasticity (18). Monocytes are classically categorized into three subgroups: CD14^++^CD16^−^ (classical), CD14^++^CD16^+^ (intermediate), and CD14^+^CD16^++^ (non-classical) (19, 20). Monocytes can also be classified into M1 (pro-inflammatory) and M2 (anti-inflammatory/pro-restorative) subtypes, based on their differentiation status. M1 monocytes highly express HLA-DR and CD86 on their surface, and secrete pro-inflammatory TNF-α and IL-6 as the dominant cytokines, whereas M2 monocytes overexpress CD163 and MerTK and mainly secrete anti-inflammatory factors such as IL-10 (17). Therefore, monocyte plasticity could be a potential therapeutic target for immune regulation in ACLF. Nonetheless, the effect of G-CSF on monocytes in ACLF patients warrants further investigation.

In Asia, the main etiology of ACLF is chronic hepatitis B virus (HBV) infection (21–23). The purpose of our study was to provide new data on the treatment of HBV-related ACLF (HBV-ACLF) using G-CSF. Here, we discuss the efficacy and safety of G-CSF, investigate the effect of G-CSF on monocytes in HBV-ACLF patients, and explore the underlying mechanisms of action of G-CSF.

## Patients and Methods

The current study includes a clinical trial and basic experiments. The former was a randomized, controlled, open-label trial to evaluate the efficacy of G-CSF for HBV-ACLF. Then, we evaluated the effect of G-CSF on the phenotype and function of monocytes from patients with HBV-ACLF.

### Patients

In the clinical trial, all participants were recruited from the Fifth Medical Center of the People’s Liberation Army (PLA) General Hospital for screening from June 2014 to September 2016. Eligible patients met the diagnostic criteria for ACLF, suggested by the Asian Pacific Association for the Study of the Liver (APASL) (1, 24), which are: (i) pre-existing diagnosed or undiagnosed chronic liver disease; (ii) acute deterioration with exacerbating jaundice (serum total bilirubin ≥ 5 mg/dL); (iii) international standard ratio (INR) ≥ 1.5 or plasma prothrombin activity (PTA; <40%); and (iv) comply with ascite and/or encephalopathy within 4 weeks. The inclusion criteria were as follows: (i) 18–70-year-old male or female; and (ii) serum hepatitis B surface antigen or HBV DNA was detected for at least 6 months. The exclusion criteria were as follows: (i) super-infection or co-infection with other hepatotropic and non-hepatotropic virus; (ii) a previous application of any immune modulator or cytotoxic/immunosuppressive therapy within the previous 12 months; (iii) hepatocellular carcinoma or extrahepatic malignancy; (iv) co-existence of severe renal, lung, brain,or heart disease or other liver disease such as alcoholic liver disease, Wilson disease, drug-induced liver injury, or autoimmune hepatitis; (v) presence of sepsis; (vi) malignant jaundice leaded by obstructive or hemolytic jaundice; and (vii) prolonged prothrombin time due to hematologic system disease.

Ascites was identified by clinical manifestations, diagnostic paracentesis, and abdominal imaging examination; Acute kidney injury was diagnosed according to the International Club of Ascites (ICA) corresponding standard (25); A West Haven classification was applied in the diagnosis of hepatic encephalopathy (26); Corresponding formulas were applied for calculation of the model for end-stage liver disease (MELD), MELD-sodium (MELD-Na), and chronic liver failure-sequential organ failure assessment (CLIF-SOFA) scores (3, 27, 28).

Diagnosis of infection was made based on clinical presentation, laboratory values and imaging examination. (i) spontaneous bacterial peritonitis: polymorphonuclear cell count in ascitic fluid ≥250/mm^3^. (ii) pneumonia: pulmonary imaging changes (infiltration, consolidation, or cavitation) plus at least 1 of the following presentation (fever ≥38°C, leucocyte count >12,000/mm^3^ or <4,000/mm^3^) plus at least 1 of the following symptoms or signs (new cough, sputum production, dyspnea, rales or bronchial breath sounds, etc.) and/or etiological evidence. (iii) urinary tract infection: Patient had at least 1of the following clinical presentations (fever, urinary tract irritation or suprapubic tenderness, etc.) and positive urine culture; or at least 2 of the above presentations and more than 10 leukocytes/μL in urine. (iv)bacteremia: positive blood culture (29, 30).

### Study Design and Follow-Up of the Clinical Trial

PASS 11.0 software (NCSS, Kaysville, UT, USA) was used to calculate the sample size. Based on our previous study, we expected that G-CSF therapy could improve the survival rate at 180 days by approximately 20% in the G-CSF group compared to that in the control group. With a statistical power of 80%, we required 52 patients in each group to detect this meaningful difference at a significance level of 5%. Considering the possible loss of patients to follow-up (10%), 114 patients were included in this study. The computer-generated randomization number code was prepared for each patient. The patients were randomly allocated to the G-CSF group (receive G-CSF therapy plus standard medical therapy) or control group (only receive standard medical therapy).

G-CSF (SL Pharm, Beijing, China) was injected subcutaneously at a dose of 5 μg/kg every day for 6 days and then every other day until day 18 (total 12 doses) in G-CSF group patients; thereafter, physical examination and laboratory tests were conducted. All patients received standard medical therapy, including intensive care monitoring, antiviral therapy, supportive therapy, and prevention and treatment for complications. All patients with infection were treated with antibiotics. Patients received albumin, terlipressin, and so on if required.

**Figure 1:**
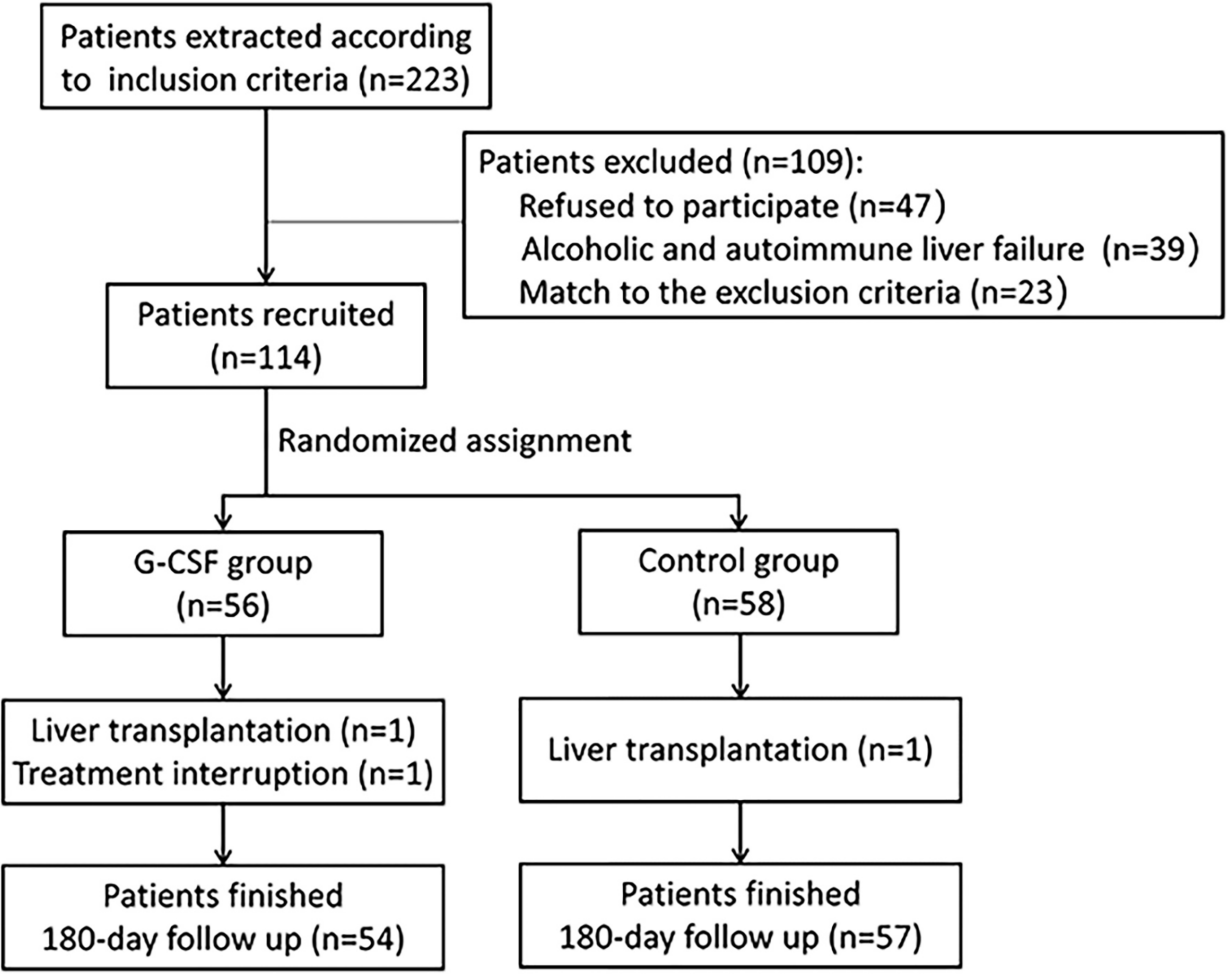
Flowchart of screening and recruitment of patients with HBV-ACLF.

A total of 114 patients meeting the inclusion and exclusion criteria were enrolled in the study (**Figure 1**). Of these, 56 patients received both standard medical therapy and G-CSF treatment (G-CSF group), and 58 patients received standard medical therapy only (control group). All patients were followed up for at least 180 days after treatment commencement. During the follow-up period, one patient in the G-CSF group discontinued treatment, and one patient each in the G-CSF group and control group underwent liver transplantation. Thus, 111 patients were included in the final analysis.

Peripheral blood samples were obtained from the enrolled patients and loaded into 10 mL heparin anticoagulant tubes for the following experiments.

### Phenotyping of Monocytes and Measurement of Cytokine Expression in G-CSF-Treated Patients

Peripheral blood mononuclear cells (PBMCs) were obtained from patients with HBV-ACLF (n = 12), before and after G-CSF treatment, to evaluate the effect of G-CSF on monocytes. Surface markers and intracellular cytokine expression in monocytes, expressed as frequency or mean fluorescence intensity (MFI), were measured using flow cytometry on the BD FACSymphony A5 analyzer (BD Biosciences, UK). Data were analyzed using the FlowJo software (Tristar, San Carlos, CA, USA).

For surface marker staining, APC-Cy7-conjugated anti-CD45 (BD Biosciences), PerCP-Cy5.5-conjugated anti-CD14 (BD Biosciences), APC-conjugated anti-CD16 (BD Biosciences), BV711-conjugated anti-HLA-DR (BD Biosciences), PE-conjugated anti-MerTK (R&D Systems), PE-Cy7-conjugated anti-CD163 (eBioscience), BV421-conjugated anti-CCR2 (BioLegend), FITC-conjugated anti-CX3CR1 (BioLegend), and BUV-737-conjugated anti-CD86 (BD Biosciences) antibodies were used. For intracellular cytokine staining (ICCS), BV-421-conjugated anti-IL-6 (BD Biosciences), PE-CF594-conjugated anti-IL-10 (BD Biosciences), and BV-510-conjugated anti-TNF-α (BioLegend) were used. Dead cells were excluded using Fixable Viability Stain 440UV (BD Bioscience).

For surface marker labeling, PBMCs were incubated with surface fluorescent-labeled monoclonal antibodies. For intracellular staining, PBMCs were incubated with or without 100 ng/mL lipopolysaccharide (LPS) and protein transport inhibitor (1μL/mL, BD GolgiPlug) for 6 h, followed by staining with surface markers, and fixation, permeabilization, and staining with the corresponding fluorescent intracellular antibodies.

### Effect of G-CSF on Monocyte Phenotype and Cytokine Secretion *In Vitro*

PBMCs were obtained from patients with HBV-ACLF who did not receive G-CSF therapy. CD14^+^ monocytes were further isolated using magnetic bead separation (Miltenyi, Bergisch Gladbach, Germany). The purity of monocyte separation was examined using flow cytometry, and CD14^+^ monocytes with a purity >95% were used *in vitro* experiments. Isolated monocytes were incubated in complete media with 20 ng/mL G-CSF or an equivalent volume of PBS (control) for 24 h for surface staining. For intracellular staining, the isolated CD14^+^ monocytes were stimulated with G-CSF (20 ng/ml) for 18 h. After 18 h incubation, LPS (100 ng/ml) and protein transport inhibitor (1μL/mL, BD GolgiPlug) was added and continue to incubation for 6 h, followed by staining with surface markers, and fixation, permeabilization, and staining with the corresponding fluorescent intracellular antibodies. After all the above steps, the monocytes were harvested and analyzed for their surface phenotype and intracellular cytokine levels using flow cytometry. Staining was performed as previously described.

### Phagocytosis and Oxidative Burst Assays

The effect of G-CSF therapy on monocyte phagocytosis and the oxidative burst capacity of patients with HBV-ACLF was evaluated. Monocyte phagocytosis and oxidative burst capacity were tested using the PHAGOTEST kit (CELONIC, Germany) and PHAGOBURST kit (CELONIC, Germany) following the manufacturer’s protocol, respectively, and then assessed by flow cytometry.

### Ethics

The study protocol and informed consent form were approved by the Human Ethics Committee of the Fifth Medical Center of the PLA General Hospital. All the procedures were in accordance with the ethical guidelines of Declaration of Helsinki. Written informed consent was obtained from patients or their guardians before enrollment. This trial was registered at ClinicalTrials.gov (NCT02331745).

### Statistical Analysis

In the clinical trial, continuous variables with normal distribution and skewed distribution were expressed as mean ± standard deviation (SD) and median [interquartile range (IQR)], respectively. Categorical variables were expressed as numbers and percentages. The chi-square test (for categorical variables) and Student’s *t*-test (for continuous variables, normal distribution) or Mann–Whitney *U* test (for continuous variables, skewed distribution) were used to compare differences between the two groups. Patients lost to follow-up contributed to the censored data. Survival rates were calculated using the Kaplan–Meier method and compared using the Log-Rank test. In the crude model and three multivariate adjusted models, Cox proportional hazards model was used for multivariate regression analysis to estimate the hazard ratios (Hazard Ratio, HRs) and 95% confidence intervals (Confidence Interval, CIs) for the risk of 180-day mortality. Statistical analyses and graphing were conducted using the statistical packages R version 3.4.3 (The R Foundation, Vienna, Austria), EmpowerStats (X&Y Solutions, Inc., Boston, MA, USA) and the MedCalc 15.2.2 (Ostend, Belgium). Statistical significance was set at *P* < 0.05.

In the Experimental section, statistical analysis was performed using GraphPad Prism 7.00 (GraphPad Software Inc., La Jolla, CA, USA). Data are presented as median (IQR). Non-parametric analyses, such as Kruskal–Wallis and Mann–Whitney *U* tests, were applied to comparisons across the different groups. Comparisons within the same individual were performed using Wilcoxon’s matched-pair test. Statistical significance was set at *P* < 0.05.

## Results

### Baseline Clinical Characteristics of Enrolled Patients

As shown in **Table 1**, the median age was 43.9 years [n = 91 men (82.0%) and 20 women (18.0%)]. Seventy patients (63.1%) patients had liver cirrhosis. MELD, MELD-Na and CLIF-SOFA scores were 23.7 (21.0–26.4), 22.9 (17.6–29.2), and 7.2 ± 1.0, respectively. The most common complication was ascites (88.3%), followed by infection (36.9%). Serum bilirubin and CRP in the G-CSF group were higher than those in the control group, but the differences were not statistically significant (*P* = 0.066 and 0.051, respectively). Meanwhile, the serum creatinine level in the control group was slightly higher than that in the G-CSF group, but both were within the normal range, and no statistical difference was detected (*P* = 0.090). **Table 1** shows that there were no significant differences in the patients’ demographic and clinical characteristics between the control and G-CSF groups.

**Table 1:** Demographical and clinical characteristic of patients in control and G-CSF Group.

Variable	Total	G-CSF group	Control group	p-value
No. of patients	111	54	57	
Age (year)	43.9 ± 10.4	42.5 ± 10.2	45.3 ± 10.6	0.154
Male, n (%)	91 (82.0%)	44 (81.5%)	47 (82.5%)	0.894
Liver Cirrhosis, n (%)	70 (63.1%)	33 (61.1%)	37 (64.9%)	0.678
White Blood Cells (10^9^/L)	6.0 (4.3-8.3)	5.9 (4.1-8.3)	6.4 (4.5-8.3)	0.439
Neutrophile (10^9^/L)	3.6 (2.5-5.6)	3.5(2.5-4.9)	4.0(2.6-5.9)	0.221
Monocyte (10^9^/L)	0.6 (0.4-0.9)	0.5 (0.4-0.9)	0.6 (0.4-0.8)	0.779
Platelets(10^9^/L)	85.0 (53.0-123.5)	90.0 (55.2-133.5)	85.0 (46.0-121.0)	0.362
Albumin (g/L)	29.0 (26.0-31.0)	29.0 (27.0-33.0)	28.0 (26.0-31.0)	0.123
Alanine aminotransferase (IU/L)	125.0 (64.0-314.5)	111.0 (62.5-300.0)	143.0 (75.0-316.0)	0.440
Aspartate transaminase (IU/L)	149.5 (90.2-285.8)	150.0 (94.0-250.0)	149.0 (89.0-288.0)	0.928
Total bilirubin (μmol/L)	291.0 (214.5-391.2)	324.4 (244.9-395.1)	273.0 (190.3-377.5)	0.066
Creatinine (μmol/L)	84.0 (72.0-97.5)	83.0 (69.2-92.0)	85.0 (74.0-108.0)	0.090
Triglycerides (mmol/L)	1.0 (0.6-1.4)	1.2 (0.7-1.6)	0.8 (0.6-1.3)	0.124
Sodium (mmol/L)	135.1 ± 4.3	135.4 ± 4.3	134.7 ± 4.3	0.373
Prothrombin activity (%)	37.2 (30.5-43.3)	38.0 (29.3-43.3)	36.4 (30.9-43.3)	0.786
International normalized ratio	1.9 (1.6-2.1)	1.8 (1.6-2.1)	1.9 (1.7-2.2)	0.263
C- reactive protein (mg/L)	12.8 (8.0-20.6)	15.6 (8.6-22.5)	10.0 (8.1-14.2)	0.051
HBV DNA (Log10 IU/mL)	4.0 (2.0-5.2)	3.8 (1.9-5.5)	4.0 (2.4-4.9)	0.674
Ascites, n (%)	98 (88.3%)	45 (83.3%)	53 (93.0%)	0.114
Infection, n (%)	41 (36.9%)	18 (33.3%)	23 (40.4%)	0.444
Acute kidney injury, n (%)	13 (11.7%)	5 (9.3%)	8 (14.0%)	0.434
Hepatic encephalopathy, n (%)	18 (16.2%)	8 (14.8%)	10 (17.5%)	0.697
MELD	23.7 (21.0-26.4)	22.8 (20.7-26.0)	24.1 (21.6-27.1)	0.261
MELD Na	22.9 (17.6-29.2)	21.8 (16.8-25.6)	23.7 (17.8-31.2)	0.152
CLIF-SOFA	7.2 ± 1.0	7.1 ± 0.9	7.3 ± 1.1	0.335

All data were present as mean ± SD, median (IQR) or number (%). G-CSF, Granulocyte-colony stimulating factor; MELD, model for end-stage liver disease; MELD-Na, MELD-sodium; CLIF-SOFA, chronic liver failure-sequential organ failure assessment.

### Adverse Effects and Main Complications During the First Month Follow-Up

The patients tolerated the treatment well, and no severe side effects were observed. One of the patients developed a mild rash during the follow-up and discontinued all drugs, including G-CSF. However, no evidence was shown that the rash was induced by G-CSF. The results showed that the G-CSF treatment protocol was safe.

During the first month, the main new-onset complications were infection, hepatic encephalopathy, and acute kidney injury (**Supplementary Table 1**). The frequency of complications was not significantly different (*P* > 0.05) between the control and G-CSF groups.

### Kaplan–Meier Comparative Survival Analysis of the Control and G-CSF Group

**Figure 2:**
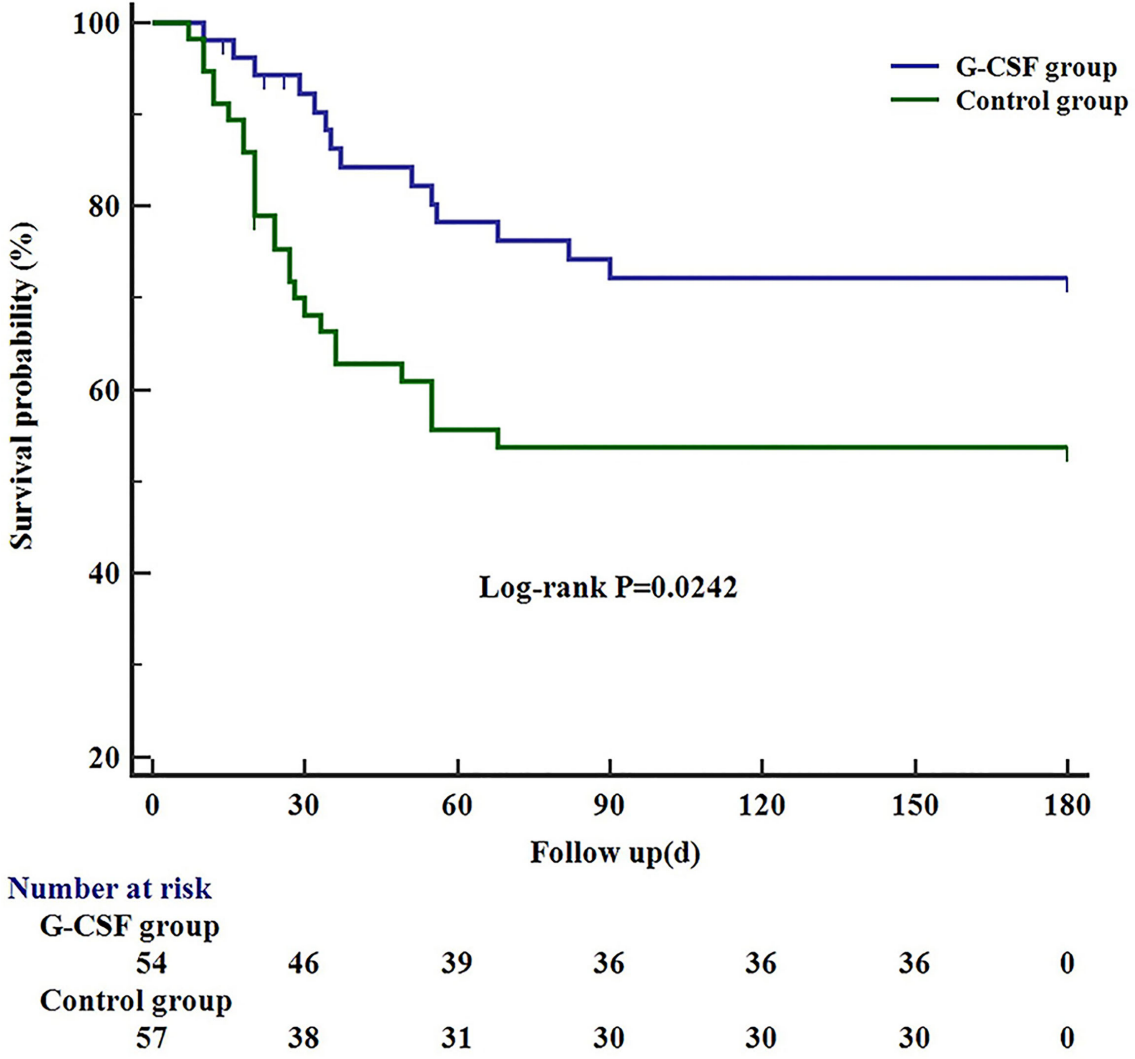
Kaplan–Meier curve showing the 180-day survival in G-CSF group, compared with the control group. G-CSF, Granulocyte-colony stimulating factor.

Of the 111 patients, 66 survived, 40 died, and 5 lost to 180 days of follow-up; thus, the survival probability at day 180 was 62.6%. In the G-CSF group, 36 patients survived, 14 patients died, and 4 patients lost to follow-up; hence, the survival probability at day 180 was 72.2%. In the control group, 30 patients survived, 26 patients died, and 1 patient lost to follow-up, with a survival probability of 53.8%. The differences between the two groups were statistically significant (*P* = 0.0242; **Figure 2**).

### Association Between Monocyte Count and 180-Day Mortality in HBV-ACLF Patients Treated With or Without G-CSF

We further explored the association between baseline and day 7 monocyte count and 180-day mortality in HBV-ACLF patients treated with or without G-CSF. As shown in **Table 2**, in both the crude and adjusted models (Models 1, 2, and 3), the baseline monocyte count was positively correlated with the 180-day mortality risk of HBV-ACLF [HR: 2.90 (1.41, 5.93), *P* = 0.0036 in Model 3], and the stratification analyses showed that this positive correlation was mainly contributed by patients in the G-CSF group [HR: 15.48 (3.60, 66.66), *P* = 0.0002 in Model 3]. However, after six consecutive days of treatment, the control group did not demonstrate significant changes, whereas the positive association between monocyte count on day 7, and the risk of death was significantly weakened in the G-CSF-treated group [HR: 1.10 (0.50, 2.43), *P* = 0.8080 in Model 3]. Similar relationship was observed between monocyte count and 90-day mortality (**Supplementary Table 2**). Therefore, we speculate that G-CSF may benefit patients with HBV-ACLF by altering the number or function of monocytes.

**Table 2:** Association between monocyte count and 180-day mortality in HBV-ACLF patients.

Model	Total	G-CSF group	Control group
Monocytes on day 0 (×10^9^/L)			
Crude model	2.42 (1.38, 4.24) 0.0020	5.19 (2.10, 12.82) 0.0004	1.72 (0.81, 3.65) 0.1599
Model 1	2.83 (1.58, 5.07) 0.0005	5.41 (2.10, 13.89) 0.0005	2.13 (0.94, 4.83) 0.0690
Model 2	2.86 (1.59, 5.12) 0.0004	5.25 (2.05, 13.46) 0.0006	2.32 (1.05, 5.12) 0.0365
Model 3	2.90 (1.41, 5.93) 0.0036	15.48 (3.60, 66.66) 0.0002	2.43 (0.72, 8.20) 0.1531
Monocytes on day 7 (×10^9^/L)			
Crude model	1.79 (1.11, 2.90) 0.0180	1.30 (0.69, 2.45) 0.4117	3.09 (1.42, 6.71) 0.0044
Model 1	1.93 (1.17, 3.19) 0.0106	1.27 (0.64, 2.51) 0.4972	3.36 (1.44, 7.85) 0.0051
Model 2	1.96 (1.17, 3.26) 0.0102	1.25 (0.63, 2.47) 0.5177	3.73 (1.58, 8.82) 0.0027
Model 3	1.42 (0.77, 2.61) 0.2590	1.10 (0.50, 2.43) 0.8080	2.57 (0.79, 8.44) 0.1184

Data are presented as HR (95% CI) and P value. Model 1 was adjusted for age and sex; Model 2 was adjusted for Model 1+ liver cirrhosis; Model 3 was adjusted for Model 2+ total bilirubin and international normalized ratio, and infection, acute kidney injury, and hepatic encephalopathy presence. G-CSF, Granulocyte-colony stimulating factor.

### Shift in the Monocyte Subpopulations After G-CSF Treatment

**Figure 3:**
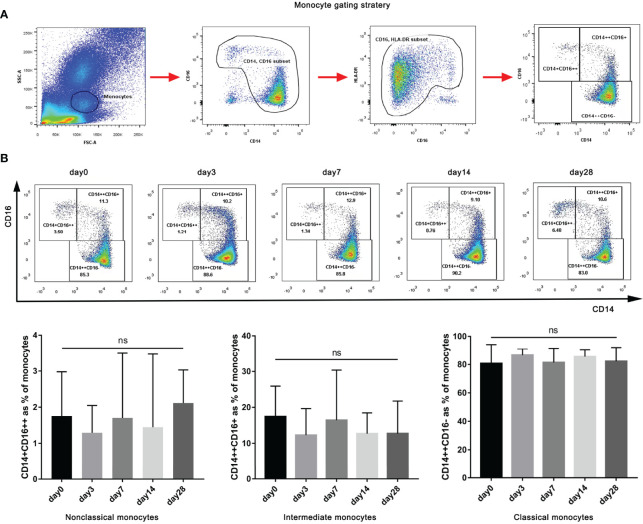
Gating of monocytes and effect of G-CSF on monocyte subtypes in patients with HBV-ACLF. **(A)** Flow cytometry analysis and gating strategy used to determine monocytes and their subsets. **(B)** Effect of G-CSF on monocyte subtypes in patients with HBV-ACLF (n=12). Non-parametric (Wilcoxon’s matched-pair test) statistical analysis was used. Data presented as median with interquartile range. ns represents *P >*0.05; G-CSF, Granulocyte-colony stimulating factor; HBV-ACLF, hepatitis B virus-related acute-on -chronic liver failure.

According to traditional gating, monocyte subtypes can be identified by the expression of CD14 and CD16 (**Figure 3A**). We evaluated the frequencies of the three monocyte subsets (described above). As shown in **Figure 3B**, although there was no significant difference in the proportion of classic, intermediate, and non-classical monocytes before (day 0) and after G-CSF treatment (*P =* 0.8981, 0.7113 and 0.9953, respectively), the intermediate and non-classical monocytes demonstrated a decreasing trend, whereas classical monocytes demonstrated an increasing trend. Thus, a shift in monocyte subpopulations was detected after G-CSF treatment.

### G-CSF Therapy Induces an Anti-Inflammatory/Pro-Restorative (M2-Like) Monocyte Phenotype in HBV-ACLF Patients

**Figure 4:**
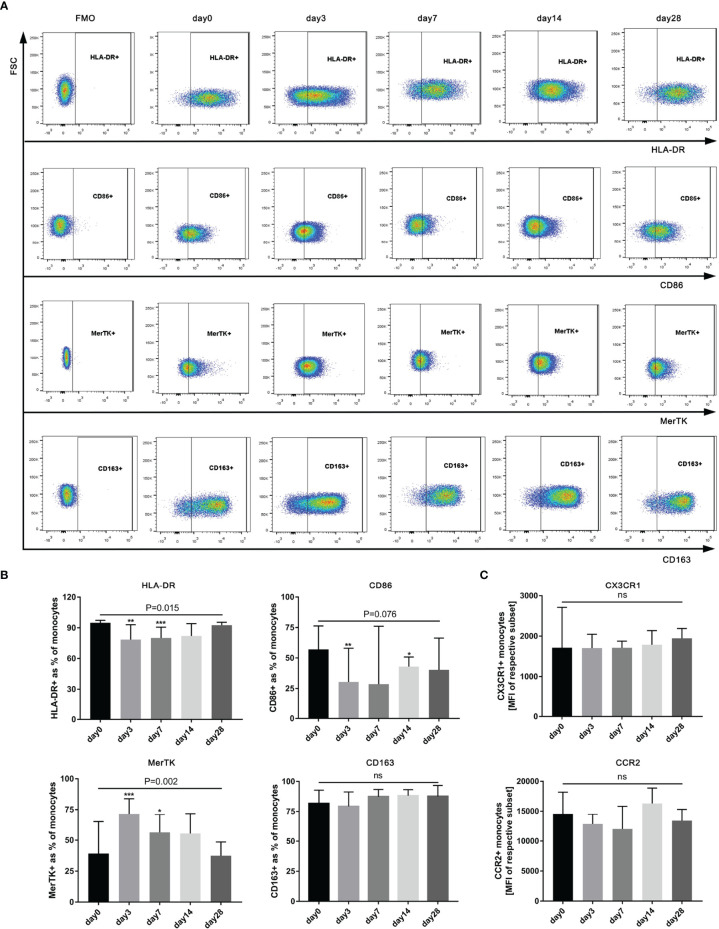
Phenotype of circulating monocytes in HBV-ACLF patients (n=12) before (day 0) and after G-CSF treatment. **(A)** Expression of CD86、HLA-DR、MerTK, and CD163 on monocytes in HBV-ACLF before and after G-CSF treatment. **(B)** Phenotypic alterations on monocytes after treated with G-CSF. **(C)** Expression of tissue-homing receptors on monocytes after treated with G-CSF. Non-parametric (Wilcoxon’s matched-pair test) statistical analysis was used. Data presented as median with interquartile range. Compared with day 0, *P < 0.05, **P < 0.01, ***P < 0.001. ns represents *P >*0.05. G-CSF, Granulocyte-colony stimulating factor; HBV-ACLF, hepatitis B virus-related acute-on -chronic liver failure; FSC, forward scatter.

To fully evaluate the effect of G-CSF on monocytes in HBV-ACLF, we designed detailed immunophenotypic analyses to detect the levels of activation/inflammation markers (HLA-DR, CD86, MerTK, and CD163) and tissue-homing markers (CCR2 and CX3CR1) before (day 0) and after the administration of G-CSF (n = 12). As shown in **Figure 4**, after treatment with G-CSF, the expression of M1-like markers (HLA-DR and CD86) in monocytes decreased (HLA-DR: day 0 vs. day 3 vs. day 7 vs. day 14 vs. day 28 = 94.9% vs. 78.5% vs. 80.1% vs. 82.1% vs. 92.6%, respectively; *P* = 0.0148; CD86: day 0 vs. day 3 vs. day 7 vs. day 14 vs. day 28 = 57.1% vs. 30.5% vs. 28.6% vs. 43% vs. 40.3%, respectively; *P* = 0.0764), whereas the expression of M2-like marker (MerTK) increased (day 0 vs. day 3 vs. day 7 vs. day 14 vs. day 28 = 39.3% vs. 71.5% vs. 56.3% vs. 55.4% vs. 37.5%, respectively; *P* = 0.0002). There was no significant change in the expression of CCR2 and CX3CR1 in monocytes according to MFI before and after G-CSF treatment (*P* = 0.1074 and 0.8889, respectively). The above results indicate that circulating monocytes after G-CSF therapy showed an anti-inflammatory/pro-restorative phenotype in patients with HBV-ACLF.

### G-CSF Therapy Attenuated Cytokine Secretion in Monocytes Obtained From HBV-ACLF Patients With or Without LPS Stimulation

**Figure 5:**
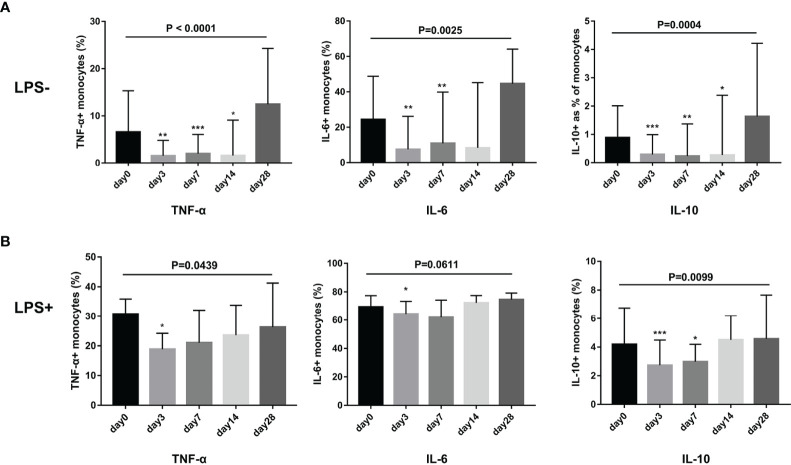
G-CSF therapy attenuated cytokine secretion in monocytes with or without LPS stimulation in HBV-ACLF patients (n=12). **(A)** Cytokine secretion in monocytes without LPS stimulation. **(B)** Cytokine secretion in monocytes with LPS stimulation. Non-parametric (Wilcoxon’s matched-pair test) statistical analysis was used. Data presented as median with interquartile range. Compared with day 0, *P < 0.05, **P < 0.01, ***P < 0.001. G-CSF, Granulocyte-colony stimulating factor; HBV-ACLF, hepatitis B virus-related acute-on-chronic liver failure; LPS, lipopolysaccharide.

We evaluated whether G-CSF treatment in patients with HBV-ACLF modulated the ability of monocytes to produce IL-6, TNF-α, and IL-10 following LPS challenge or not. As shown in **Figure 5**, the secretion of TNF-α, IL-6, and IL-10 by monocytes decreased after G-CSF therapy without LPS stimulation (TNF-α: day 0 vs. day 3 vs. day 7 vs. day 14 vs. day 28 = 6.6% vs. 1.6% vs. 2.0% vs. 1.6% vs. 12.5%, respectively; *P* < 0.0001; IL-6: day 0 vs. day 3 vs. day 7 vs. day 14 vs. day 28 = 24.0% vs. 7.5% vs. 10.9% vs. 8.4% vs. 44.6%, respectively; *P* = 0.0025; IL-10: day 0 vs. day 3 vs. day 7 vs. day 14 vs. day 28 = 0.9% vs. 0.5% vs. 0.2% vs. 0.5% vs. 1.6%, respectively; *P* = 0.0004). In contrast to pre-treatment (day 0), cytokine secretion in monocytes showed a decreased response to LPS stimulation after G-CSF treatment (TNF-α: day 0 vs. day 3 vs. day 7 vs. day 14 vs. day 28 = 30.8% vs. 18.8% vs. 21.0% vs. 23.6% vs. 26.3%, respectively; *P* = 0.0439; IL-6: day 0 vs. day 3 vs. day 7 vs. day 14 vs. day 28 = 69.4% vs. 64.5% vs. 61.8% vs. 72.3% vs. 74.7%, respectively; *P* = 0.0611; IL-10: day 0 vs. day 3 vs. day 7 vs. day 14 vs. day 28 = 4.2% vs. 2.7% vs. 3.0% vs. 4.5% vs. 4.6%, respectively; *P* = 0.0099). Hence, G-CSF reduces the inflammatory response of monocytes in HBV-ACLF patients.

### G-CSF Induces M2-Like Phenotype and Functional Transition of Monocytes From HBV-ACLF Patients *In Vitro*

**Figure 6:**
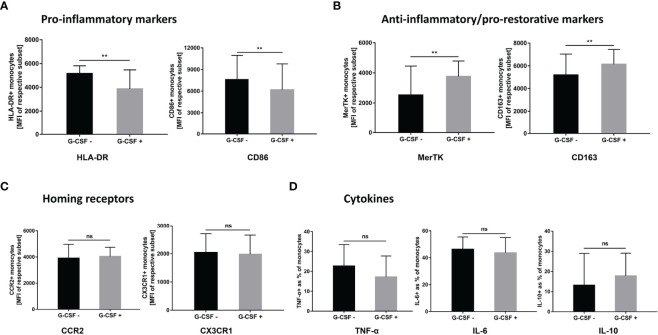
G-CSF induces M2-like phenotype and functional transition of monocytes from HBV-ACLF patients *in vitro*. **(A)** G-CSF decreased the expression of pro-inflammatory markers on monocytes (n=9). **(B)** G-CSF elevated the expression of anti-inflammatory/pro-restorative markers on monocytes (n=9). **(C)** Effect of G-CSF on the expression of homing receptors on monocytes (n=9). **(D)** G-CSF attenuated pro-inflammatory cytokine secretion in monocytes (n=5). Non-parametric (Wilcoxon’s matched-pair test) statistical analysis was used. Data presented as median with interquartile range. ** represents compared with day 0, *P*<0.01; ns represents *P >*0.05; G-CSF, Granulocyte-colony stimulating factor; HBV-ACLF, hepatitis B virus-related acute-on -chronic liver failure.

We performed *in vitro* experiments to further clarify the effect of G-CSF on the phenotype and function of monocytes in patients with HBV-ACLF. As shown in **Figure 6**, after exogenous addition of G-CSF to monocytes from HBV-ACLF patients, the expression of M1-type markers (HLA-DR and CD86) decreased, whereas the expression of M2-type markers (CD163 and MerTK) increased (*P* < 0.01). There was no significant difference in the expression of homing receptors CCR2 and CX3CR1 between the two groups (*P* > 0.05; **Figure 6C**). Concurrently, although no statistically significant difference was detected, G-CSF decreased the secretion of pro-inflammatory factors (IL-6 and TNF-α) after LPS stimulation, whereas IL-10 secretion was slightly increased (**Figure 6D**). Except for the secretion of IL-10, these results were consistent with those observed *in vivo*. G-CSF tend to promote the transition of monocytes to M2 type in HBV-ACLF patient.

### Influence of G-CSF on Phagocytosis and Oxidative Burst Function of Monocytes in HBV-ACLF

**Figure 7:**
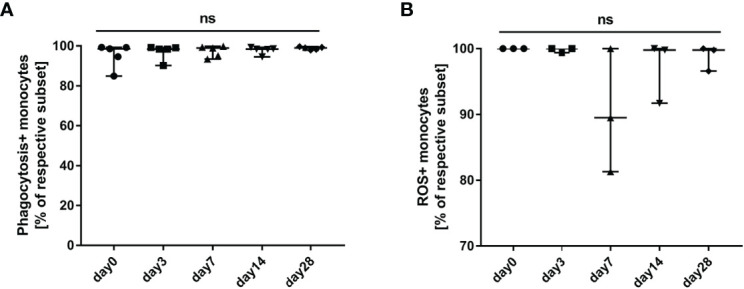
Influence of G-CSF on phagocytosis and oxidative burst function of monocytes in HBV-ACLF. **(A)** Effect of G-CSF on phagocytosis function of monocytes (n=5). **(B)** Effect of G-CSF on oxidative burst function of monocytes (n=3). ns represents *P >*0.05; G-CSF, Granulocyte-colony stimulating factor; HBV-ACLF, hepatitis B virus-related acute-on -chronic liver failure.

We tested and compared phagocytosis (**Figure 7A**) and oxidative burst (**Figure 7B**) of monocytes freshly isolated from HBV-ACLF patients before (day 0) and after G-CSF treatment. It was observed that phagocytosis of monocytes showed an upward trend, whereas oxidative burst showed a downward trend after the administration of G-CSF; however, the discrepancies were not statistically significant in view of the small numbers (*P* all <0.05).

## Discussion

In the present study, we conducted a randomized clinical trial to treat HBV-ACLF patients with G-CSF. We showed that G-CSF significantly improved the survival of HBV-ACLF patients. In addition, Cox regression analysis showed that G-CSF treatment attenuated the positive correlation coefficient between monocyte count and 180-day mortality risk in patients with HBV-ACLF. Further analyses demonstrated that after treatment with G-CSF, the phenotype and function of monocytes in HBV-ACLF tended to an anti-inflammatory/pro-restorative (M2-like) phenotype, which may contribute to the attenuation of liver injury and promote recovery of ACLF. These novel findings expand our knowledge of the role of G-CSF in the treatment of liver failure and provide new evidence that G-CSF may contribute to protection against liver damage.

In the current study, we observed a survival benefit in patients with HBV-ACLF after G-CSF therapy. This result was in agreement with those of several previous studies (5, 6). A study performed by Garg et al. (6) showed that the 60-day survival rate was significantly higher in ACLF patients treated with G-CSF than in the placebo group (69.6% vs. 29.2%; *P* = 0.001). Another study also revealed superior 90-day survival (5) of HBV-ACLF patients treated with G-CSF compared to those treated with the placebo (64.3% vs. 28.6%; *P* = 0.04). In addition, these two studies showed improvement in liver function, as indicated by the MELD and Child-Turcotte-Pugh (CTP) scores. However, a European study by Engelmann et al. (10) failed to demonstrate better efficacy of G-CSF than standard medical therapy in ACLF patients (90-day transplant-free survival rates: 34.1% vs. 37.5%; *P* = 0.805). The liver function scores also did not improve in the G-CSF treatment group. The positive effect of G-CSF has only been confirmed in Asian studies, and not in the European study.

A major potential factor that may have contributed to this discrepancy is that the inclusion criteria were different. In the studies conducted by Gerg and Duan (5, 6), ACLF was defined according to the APASL criteria, in which patients with pre-exiting decompensated cirrhosis and sepsis were not included. The European study enrolled patients with acute decompensation of cirrhosis, utilizing the European Association for the Study of the Liver -Chronic Liver Failure (EASL-CLIF) criteria. APASL criteria focuses on liver failure, and severe liver necroinflammation is regarded as the core feature of ACLF. However, organ failure, whether hepatic or extrahepatic, was the predominant feature according to the EASL-CLIF criteria (31). ACLF patients, who meet EASL-CLIF criteria, are typically and more irreversibly at the “end-stage.” G-CSF is regarded as a growth factor in hepatic regeneration and an immunomodulatory agent in ACLF (2). Garg et al. (6) showed that the number of CD34^+^ cells markedly increased in the liver tissue after G-CSF therapy in patients with ACLF. Subsequently, they demonstrated that G-CSF enhanced the mobilization of bone marrow hematopoietic stem cells and promoted their homing in the hepatic parenchyma. G-CSF therapy was also shown to enhance dendritic cell recruitment to the liver and suppress interferon-γ secretion by CD8^+^ T cells to attenuate hepatocellular injury (13). The purpose of G-CSF in patients with ACLF is to enhance liver regeneration and regulate the immune and inflammatory responses. Hence, G-CSF should be used in patients with early stage ACLF, where it can offer excellent regenerative potential. However, in the study by Engelmann (10), all G-CSF-treated ACLF patients had a poor pre-existing liver base (liver cirrhosis), more than 65.9% suffered from extrahepatic organ failure, and up to 56.8% had bacterial infection at baseline. Thus, it may have been too late for G-CSF to exert a therapeutic effect. In a comment on this study, Sarin (32) offers a similar opinion. Therefore, the differences in the definition of ACLF between the East and the West also directly led to the discrepancy in understanding the pathophysiological process, therapeutic strategies, and treatment effects in ACLF.

Our study also showed that the baseline monocyte count was positively associated with the 180-day mortality risk of HBV-ACLF patients, while the stratified analysis showed that this positive correlation was mainly contributed by the patients in the G-CSF group. Interestingly, after 6 days of continuous treatment, the control group did not demonstrate significant changes, whereas the positive association between monocyte count on day 7 and the risk of death was significantly weakened in the G-CSF-treated group. Therefore, we speculate that G-CSF may benefit HBV-ACLF patients by altering the number or function of monocytes.

Monocyte/macrophage dysfunction plays a core role in the progression of ACLF (17). These cells have plastic phenotypes and diverse roles in ACLF, from pro-inflammatory (M1-like) to anti-inflammatory/pro-restorative (M2-like), depending on the microenvironment. To better understand the mechanism of action of G-CSF, we investigated its effect on the phenotype and function of monocytes in ACLF patients. Our study demonstrated that after treatment with G-CSF, the expression of M1-like markers (HLA-DR and CD86) in monocytes decreased, whereas expression of MerTK (M2-like marker) increased. Weise et al. (33) detected a similar phenomenon in mouse models of ischemic stroke. After administration of G-CSF, the expression of Ly6c^+^ (M1-like marker) in monocytes was markedly suppressed (33). Similarly, in another study performed by Fadini, circulating M2-like phenotype monocytes were evaluated after G-CSF treatment (34). Our study also showed that the secretion of TNF-α, IL-6, and IL-10 by monocytes decreased after G-CSF therapy with or without LPS stimulation in patients with ACLF. This indicates that G-CSF could attenuate the monocyte immune response in the basal state in ACLF. Moreover, the response to further LPS stimuli was reduced, which indicated LPS tolerance. Interestingly, *in vitro* experiments, we found that the IL-10 level was slightly increased by G-CSF after LPS stimulation. Weise et al. also found that G-CSF may promote the production of IL-10 (an M2-like cytokine) in monocytes in animal models of ischemic stroke (33). This discrepancy may be related to the methodological limitations of the studies. The microenvironment is also different *in vivo* vs. *in vitro*, and monocytes in patients may also be affected by other stimuli. What is more, several studies have detected an anti-inflammatory effect of monocytes mediated by G-CSF, which is characterized by the reduction of LPS-induced TNF-α (M1-like cytokine) production (35, 36). These findings appear to be compatible with our results. It means that G-CSF appears to impair antimicrobial defenses while attenuating monocyte inflammatory responses in ACLF. However, our study also found that after G-CSF treatment, the phagocytic function of monocytes in patients tended to be elevated, while the oxidative burst capacity decreased. Due to the small sample size, these differences were not statistically significant. Therefore, further studies may be required to assess whether G-CSF has a negative effect on the antimicrobial defense capacity of monocytes. Collectively, these results indicate that G-CSF seems to induce monocytes to exert immunosuppressive effects. From this perspective, it supports the argument that G-CSF should be used in the early phase of ACLF, when the pro-inflammatory/injury response is dominant (37).

Our study had several limitations. Firstly, this was a single-center study, and a larger multicenter trial should be conducted. Secondly, the relatively small sample size in the experimental study may have led to statistical fluctuations. Finally, because liver tissues were unavailable, we only investigated the effect of G-CSF on circulating monocytes in ACLF patients, and lacked macrophages. This issue will be resolved in future animal experiments.

In summary, our study validated the efficacy of G-CSF in patients with HBV-ACLF. In addition, the effects of G-CSF on monocytes in ACLF were explored. G-CSF can promote the anti-inflammatory/pro-restorative phenotype (M2-like) transition of monocytes, which may contribute to the recovery of ACLF. However, owing to heterogeneity, monocytes play distinct roles in ACLF, from pro-inflammatory to pro-resolution, depending on the microenvironment. Therefore, a full understanding of the pathophysiological processes and developmental stages of ACLF may help to select the right patients for appropriate therapy.

## Data Availability Statement

The raw data supporting the conclusions of this article will be made available by the authors, without undue reservation.

## Ethics Statement

The studies involving human participants were reviewed and approved by the Human Ethics Committee of the Fifth Medical Center of the PLA General Hospital. The patients/participants provided their written informed consent to participate in this study.

## Author Contributions

JT, HW, XX, JC (6th author), XM, ZL, HS, XL and CL participated in the data acquisition. JH, ZW, HF and JT designed the study. JT, HW and HF performed analyses and interpretation of data. JT, HW, XM, JC (9th author) and HXW completed the experiments. JT, HW, ZW wrote the first draft of the manuscript and incorporated revisions. JT, HW and JH prepared the final version. All authors contributed to the article and approved the submitted version.

## Funding

This work was supported by a grant from the Capital’s Funds for Health Improvement and Research, China (NO.2020-1-5031) and National Clinical Research Center for Infectious Diseases (NCRC-ID202106).

## Conflict of Interest

The authors declare that the research was conducted in the absence of any commercial or financial relationships that could be construed as a potential conflict of interest.

## Publisher’s Note

All claims expressed in this article are solely those of the authors and do not necessarily represent those of their affiliated organizations, or those of the publisher, the editors and the reviewers. Any product that may be evaluated in this article, or claim that may be made by its manufacturer, is not guaranteed or endorsed by the publisher.

## Abbreviations

ACLF, acute-on-chronic liver failure; G-CSF, granulocyte colony-stimulating factor; HBV-ACLF, hepatitis B virus (HBV)-related ACLF; APASL, Asian-Pacific Association for the Study of the Liver; PBMCs, peripheral blood mononuclear cells; LPS, lipopolysaccharide; MELD, Model for End-Stage Liver Disease; MELD-Na, MELD-sodium; CLIF-SOFA, Chronic Liver Failure-Sequential Organ Failure Assessment; CTP, Child-Turcotte-Pugh; EASL-CLIF, European Association for the Study of the Liver -Chronic Liver Failure; IQR, interquartile range; HR, hazard risk; CI, confidence interval.
