# Supplementary material for: Granulocyte Colony-Stimulating Factor Accelerates the Recovery of Hepatitis B Virus-Related Acute-on-Chronic Liver Failure by Promoting M2-Like Transition of Monocytes
Source: Front Immunol. 2022 May 16;13:885829. doi: 10.3389/fimmu.2022.885829 (PMC9148949; doi:10.3389/fimmu.2022.885829)
Supplement: Supplementary file 1 [file Table_1.docx]

**Supplementary Material**

**Supplementary Table 1 Main complications in enrolled patients during 1-month follow-up**

| **Main complications** | **Control group (n=57)** | **G-CSF group (n=54)** | **P-value** |
| --- | --- | --- | --- |
| Infection | 18 (24.56%) | 14 (27.77%) | 0.50 |
| Spontaneous bacterial peritonitis | 10 (17.54%) | 9 (16.98%) |  |
| Pneumonia | 6 (10.5%) | 4 (7.40%) |  |
| Fungal pneumonia | 1 (1.75%) | 0 |  |
| Oral cavity fungal infection | 0 | 1 (1.85%) |  |
| Urinary infection | 1 (1.75%) | 0 |  |
| Hepatic encephalopathy | 4 (7.01%) | 1 (1.85%) | 0.19 |
| Acute kidney injury | 4 (7.01%) | 4 (7.40%) | 0.91 |

Data were present as number (%); G-CSF: Granulocyte-colony stimulating factor;

**Supplementary Table 2. Association between monocyte count and 90-day mortality in HBV-ACLF patients.**

| **Model** | **Total** | **G-CSF group** | **Control group** |
| --- | --- | --- | --- |
| Monocytes on day 0 (×10^9^/L) |  |  |  |
| Crude model | 2.40 (1.36, 4.23) 0.0025 | 5.00 (2.00, 12.53) 0.0006 | 1.72 (0.81, 3.65) 0.1599 |
| Model 1 | 2.82 (1.56, 5.10) 0.0006 | 5.28 (2.03, 13.72) 0.0006 | 2.13 (0.94, 4.83) 0.0690 |
| Model 2 | 2.87 (1.59, 5.18) 0.0005 | 5.23 (1.99, 13.70) 0.0008 | 2.32 (1.05, 5.12) 0.0365 |
| Model 3 | 2.88 (1.39, 5.95) 0.0044 | 14.24 (3.35, 60.52) 0.0003 | 2.43 (0.72, 8.20) 0.1531 |
| Monocytes on day 7 (×10^9^/L) |  |  |  |
| Crude model | 1.88 (1.15, 3.07) 0.0114 | 1.39 (0.73, 2.64) 0.3167 | 3.09 (1.42, 6.71) 0.0044 |
| Model 1 | 2.04 (1.22, 3.40) 0.0064 | 1.40 (0.70, 2.81) 0.3408 | 3.36 (1.44, 7.85) 0.0051 |
| Model 2 | 2.09 (1.25, 3.52) 0.0053 | 1.40 (0.69, 2.80) 0.3487 | 3.73 (1.58, 8.82) 0.0027 |
| Model 3 | 1.54 (0.83, 2.86) 0.1716 | 1.20 (0.54, 2.70) 0.6543 | 2.57 (0.79, 8.44) 0.1184 |

Data are presented as HR (95% CI) and P value. Model 1 was adjusted for age and sex; Model 2 was adjusted for Model 1+ liver cirrhosis; Model 3 was adjusted for Model 2+ total bilirubin and international normalized ratio, and infection, acute kidney injury, and hepatic encephalopathy presence. G-CSF: Granulocyte-colony stimulating factor;
